# IMB-6G, a novel *N*-substituted sophoridinic acid derivative, induces endoplasmic reticulum stress-mediated apoptosis *via* activation of IRE1α and PERK signaling

**DOI:** 10.18632/oncotarget.8184

**Published:** 2016-03-18

**Authors:** Na Zhang, Chongwen Bi, Lu Liu, Yueying Dou, Sheng Tang, Weiqiang Pang, Hongbin Deng, Danqing Song

**Affiliations:** ^1^ Institute of Medicinal Biotechnology, Chinese Academy of Medical Sciences and Peking Union Medical College, Beijing 100050, China

**Keywords:** sophoridinic acid, ER stress, apoptosis, hepatocellular carcinoma, CHOP

## Abstract

Sophoridinic acid derivatives have received considerable attentions for their potencies in cancer therapy. IMB-6G is a novel N-substituted sophoridinic acid derivative with potent cytotoxicity against tumor cells. In the present study, we explored the antitumor abilities of IMB-6G in human hepatocellular carcinoma (HCC) cells and investigated the underlying mechanisms. We found that IMB-6G inhibited cell growth and induced mitochondrial-dependent apoptosis in HepG2 and SMMC7721 cells. Analyses of the molecular mechanism of IMB-6G-induced apoptosis indicated IMB-6G induced endoplasmic reticulum (ER) stress activation. Incubation of HCC cells with IMB-6G induced increase in Bip and CHOP levels, which precede induction of apoptosis. Further study showed IMB-6G activated IRE1α and PERK pathways but did not stimulated ATF6 pathway in HCC cells. Moreover, silencing of IRE1α dramatically abrogated IMB-6G-induced pro-apoptotic ASK1-JNK signaling. Importantly, interruption of CHOP rendered HCC cells sensitive to IMB-6G-induced apoptosis via inactivation of Bim, PUMA and Bax. Thus, the IRE1α-ASK1 and PERK-CHOP pathways may be a novel molecular mechanism of IMB-6G-induced apoptosis. Collectively, our study demonstrates that IMB-6G induces ER stress-mediated apoptosis by activating IRE1α and PERK pathways. Our findings provide a rationale for the potential application of IMB-6G in HCC therapy.

## INTRODUCTION

Hepatocellular carcinoma (HCC) is the third most common cause of cancer death worldwide, particularly in Africa and Asia [1]. The short-term prognosis of patients with HCC has improved recently due to advances in early diagnosis and treatment, but long-term prognosis remains unsatisfactory [2]. Therefore, the identification of new therapeutic agents is required for the effective treatment of HCC.

Endoplasmic reticulum (ER) plays important roles in regulation of lipid synthesis, calcium homeostasis, protein folding and trafficking [3]. Various cellular stresses, such as nutrient deprivation, metabolic stress, and antitumor drugs, induce ER stress and trigger an adaptive response known as the unfolded protein response (UPR) [4]. UPR is executed by the activation of three transmembrane ER sensors: PERK (pancreatic endoplasmic reticulum eIF2a kinase), IRE1 (inositol requiring enzyme 1), and ATF6 (activating transcription factor 6) [5]. ER stress maintains and restores ER homeostasis by inducing ER chaperones, such as the binding immunoglobulin protein (Bip) that mediates protein refolding [6]. Moderate ER stress is protective for cell survival, but prolonged or severe ER stress may lead to death-receptor and mitochondrial-mediated apoptosis [7]. ER stress-mediated apoptosis is associated with (i) IRE1α-mediated activation of TRAF2 (tumor necrosis factor receptor associated factor 2), which stimulates the ASK1 (apoptosis signal-regulating kinase 1)/JNK (c-Jun N-terminal kinase) cascade that promote apoptosis [8]; (ii) PERK/eIF2α-dependent induction of the proapoptotic transcriptional factor CHOP(C/EBP homologous protein), which increasing expression of Bim and PUMA, while decreasing expression of Bcl-2 [9]; and (iii) Bax/Bcl2-regulated Ca^2+^ release from the ER [10].

Cancer cells are constantly under certain levels of ER stress due to hypoxia and high loads of mutant proteins [11]. Mounting evidence suggests that increasing ER stress might be an effective strategy to eliminate cancer cells [12]. Recent studies have confirmed that anticancer agents such as Everolimus, Fucoidan and Curcumin aggravate ER stress response and activate ER stress-mediated apoptosis pathway in cancer cell [13–15]. Importantly, relieving ER stress impaired anticancer agents -induced apoptosis. Therefore, agents that induce excessive ER stress have promising antitumor effects.

**Figure 1:**
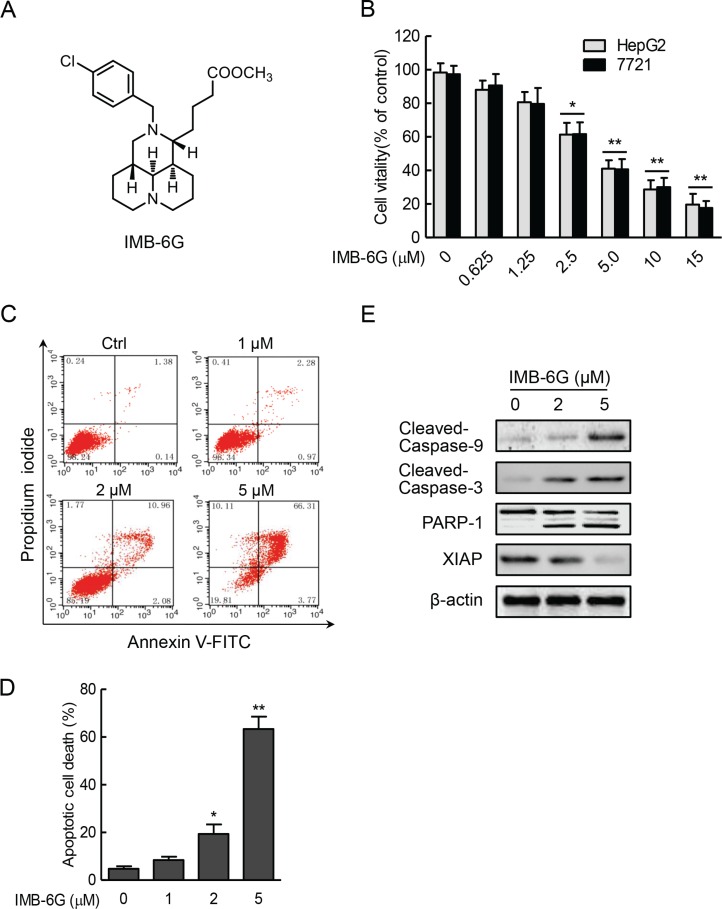
IMB-6G inhibits cell proliferation and induces apoptosis in HCC cells (**A**) Structure of the IMB-6G molecule. (**B**) HepG2 and SMMC7721 cells were treated with various concentrations of IMB-6G for 24 h, cell viability was assayed by MTT assay. (**C**) HepG2 cells were stained with Annexin V-FITC and propidium iodide (PI) after incubated with indicated concentrations of IMB-6G for 24 h, the numbers of apoptotic cells were analyzed by flow cytometry. (**D**) Statistical analysis result of flow cytometric analysis of apoptosis. Annexin V-positive cells were accepted as apoptotic cells. The results are presented as mean ± standard error and represent three individual experiments. (**E**) HepG2 cells were treated with indicated concentrations of IMB-6G for 24 h. The protein expression levels of cleaved caspase-9, cleaved caspase-3, poly (ADP-ribose) polymerases (PARP) and X-linked inhibitor of apoptosis protein (XIAP) were detected by immunoblotting. *p < 0.05, **p < 0.01 compared with the untreated control group.

Sophoridine, one of the major bioactive components extracted from the traditional medicine herb *Sophora alopecuroides* L., has been widely used as an antitumor drug against malignant trophoblastic tumors [16, 17] and a lot of attention has been drawn to further development of its analog. IMB-6G (Figure 1A) is a new *N*-substituted sophoridinic acid derivative showing a potent antiproliferation effect in a panel of human tumor cell lines via inducing G0/G1 cell cycle arrest and apoptosis [18]. Furthermore, IMB-6G also showed a reasonable bioavailability, favorable pharmacokinetic property and good safety *in vivo* [18, 19]. However, cellular and molecular mechanism underlying the antitumor effects of IMB-6G remains unknown.

In the present study, we aimed to investigate the antitumor activity and the underlying mechanisms of IMB-6G against human HCC cells. Our results indicated that IMB-6G induces apoptosis through the activation of the ER stress. Furthermore, IRE1α-ASK1 and PERK-CHOP-mediated ER stress might be involved in the signaling of IMB-6G-induced apoptosis, suggesting that IMB-6G targets ER stress and has potential as a novel chemotherapeutic agent for the treatment of HCC.

## RESULTS

### IMB-6G induces cytotoxicity and apoptosis in HCC cells

To investigate the antitumor activity of IMB-6G on HCC, human HCC cells (HepG2 and SMMC7721) were incubated for 24 hours with increasing concentrations of IMB-6G and its cytotoxic effect was determined by MTT assay. As shown in Figure 1B, IMB-6G inhibited the proliferation of HepG2 and SMMC7721 cells in a concentration-dependent manner. Statistically significant cytotoxic effects are observed at concentration above 2.5 μM (Figure 1B). To examine whether cell apoptosis was involved in IMB-6G-induced HCC cell death, Annexin V/PI double staining was used to evaluate the apoptotic cell death of IMB-6G-treated HepG2 cells. Flow cytometry results indicated that IMB-6G induced phosphatidylserine plasma membrane externalization in HepG2 cells in a dose-dependent manner (Figure 1C and 1D). Similar results were obtained in IMB-6G-treated SMMC7721 cells (Supplementary Figure S1). This effect was inhibited by Z-VAD (Supplementary Figure S2), a pancaspase inhibitor, indicating that IMB-6G induces apoptotic cell death associated with caspase activation. Furthermore, immunoblotting results (Figure 1E) also showed that IMB-6G induced the activation of caspase-9 and caspase-3, cleavage of PARP-1 and decreased the level of anti-apoptotic protein XIAP. These results thus demonstrate that IMB-6G induces cytotoxicity and apoptosis in HCC cells.

### IMB-6G induces apoptosis in HCC cells on the mitochondrial-dependent pathway

**Figure 2:**
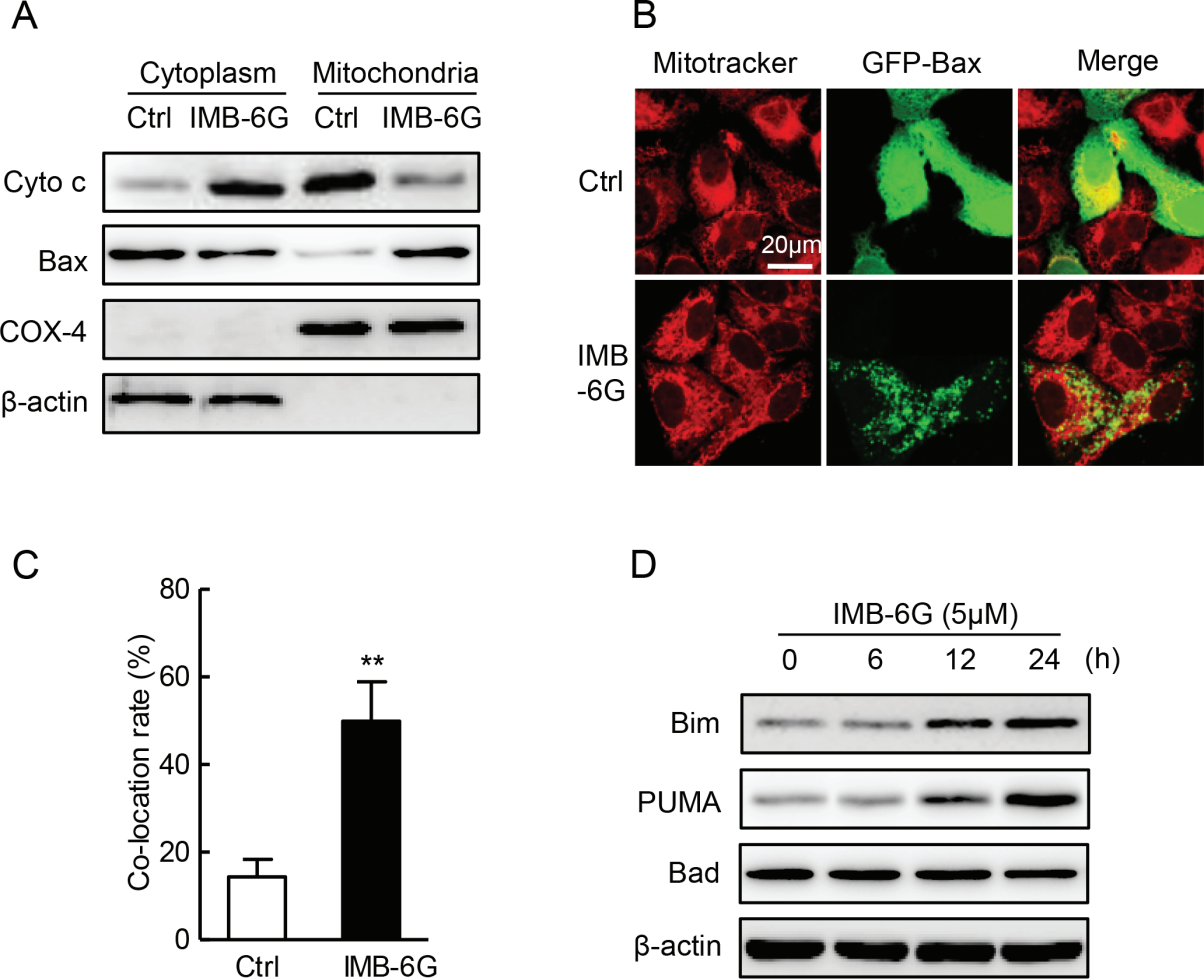
Effect of IMB-6G on the cytochrome c release, Bax translocation and BH3-only proteins (**A**) HepG2 cells were treated with or without 5 μM IMB-6G for 24 h. The levels of Cytochrome c and Bax in cytoplasm and mitochondria fraction were measured by immunoblotting. Expression of β-actin and COX-4 were used for the loading control of cytoplasm and mitochondria fractions. (**B**) HepG2 cells were transfected with GFP-Bax plasmid for 24 h, followed by 5 μM IMB-6G treatment for 16 h. The effect of IMB-6G on Bax translocation to mitochondria was visualized by confocal microscope. MitoTracker (red) was used as a mitochondria-specific marker. Merged images are shown, and the yellow color represents co-localization of Bax (green) and MitoTraker (red; scale bar, 20 μm). (**C**) Percentage of Bax translocation was determined by counting three different fields (20–40 cells/field), and the statistical analysis result is shown. **p < 0.01 compared with control group. (**D**) HepG2 or SMMC7721 cells were treated with 5 μM IMB-6G for the indicated time, the protein levels of Bim, PUMA, Bad and β-actin were measured by immunoblotting using corresponding antibodies.

The release of Cytochrome c from mitochondria to cytoplasm and the translocation of Bax from cytoplasm to mitochondria are required for caspase activation that initiates the apoptotic program [20]. To investigate whether mitochondrial-dependent apoptosis involved in IMB-6G-induced cell death, we examined the effects of IMB-6G on Cytochrome c release and Bax translocation. Immunoblotting analysis showed that the protein level of Cytochrome c dramatically decreased in the mitochondria of HepG2 cells after treatment with IMB-6G (Figure 2A). At the same time, the level of the Bax in the mitochondria was significantly increased by IMB-6G (Figure 2A). Furthermore, the translocation of Bax into the mitochondria induced by IMB-6G is clearly shown in Figure 2B and 2C. In the control cells, GFP-Bax signal (green fluorescence) was distributed diffusely in the cytoplasm. On the contrary, in IMB-6G-treated HepG2 cells, Bax became punctuate and was co-localized with mitochondria (red fluorescence). These results indicated that IMB-6G activated mitochondrial-based Bax translocation, which might induce apoptosis. Additionally, to check whether BH3-only proteins were involved in the signal transduction of IMB-6G-induced apoptosis, the expression levels of Bim, p53-upregulated modulator of apoptosis (PUMA) and Bad were checked by immunoblotting. Our results showed that IMB-6G increased the BH3-only protein levels of Bim and PUMA, but not Bad, in HepG2 and SMMC7721 cells (Figure 2D). Taken together, these data indicate that IMB-6G triggers apoptosis through the intrinsic mitochondrial-dependent pathway in HCC cells.

### IMB-6G triggers ER stress in HCC cells

**Figure 3:**
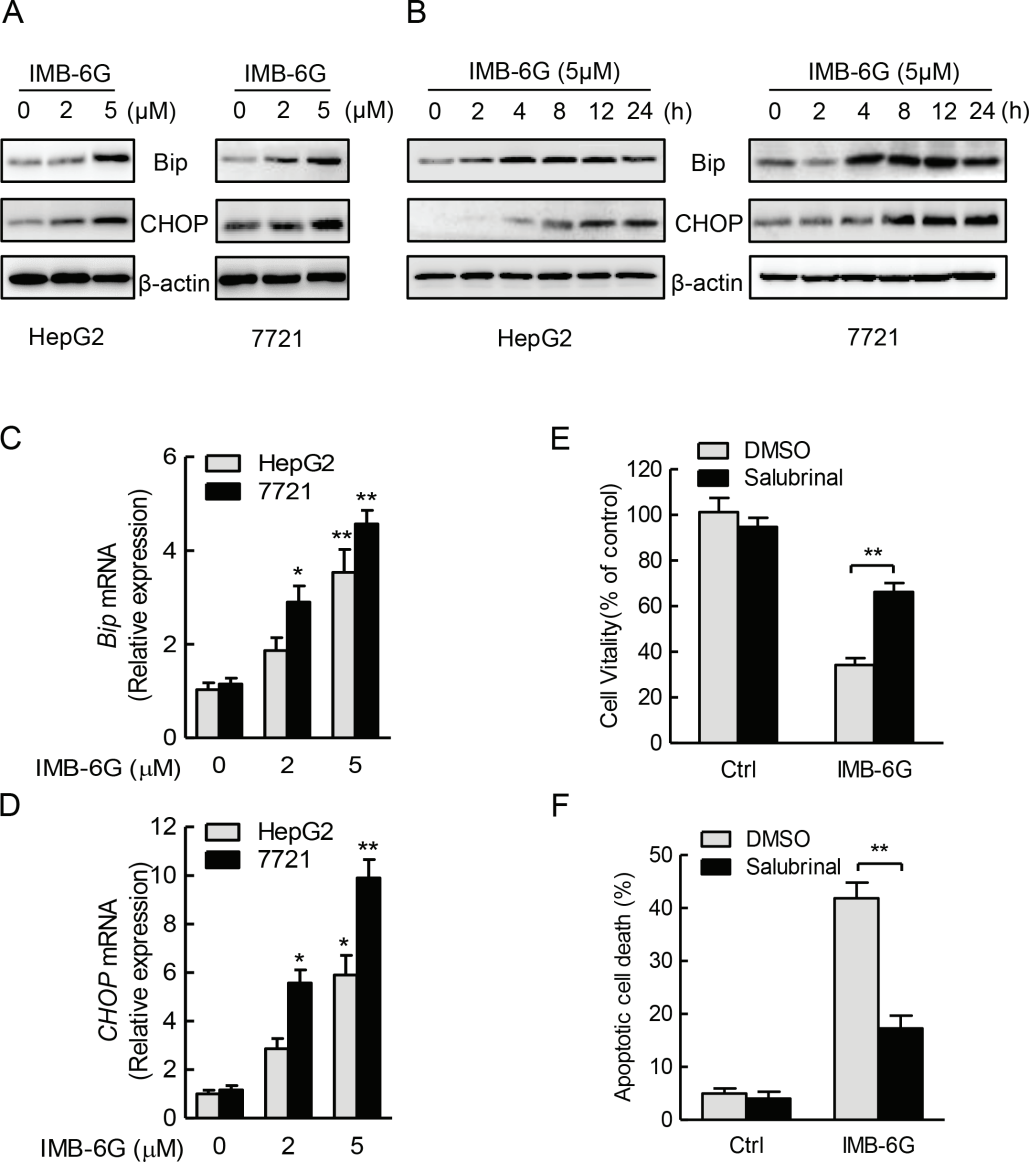
IMB-6G triggers ER stress in HCC cells (**A** and **B**) HepG2 and SMMC7721cells were treated with IMB-6G at the indicated concentrations for 24 h (A), or treated with IMB-6G (5 μM) for indicated time points (B), the expression levels of Bip, CHOP and β-actin were measured by immunoblotting using corresponding antibodies. (**C** and **D**) HepG2 and 7721cells were treated with IMB-6G at the indicated concentrations for 16 h. Total RNAs were extracted and subjected to real-time PCR analysis using specific primer sets for Bip, CHOP, and GAPDH, and the data were normalized to GAPDH expression. *p < 0.05, **p < 0.01 compared with untreated control group. (**E** and **F**) HepG2 cells were pre-treated with ER stress inhibitor salubrinal (Sal, 20 μM) for 1 h, followed by IMB-6G (5 μM) treatment for 24 h, cell viability was analyzed by MTT assay (E), and the apoptotic rate was determined by flow cytometry (F). **p < 0.01 compared with IMB-6G-treated control group.

Accumulating evidences indicate that ER stress plays a crucial role in the regulation of apoptosis [21, 22]. To confirm the hypothesis that the ER stress played a role in IMB-6G-mediated apoptosis, we analyzed ER stress responses in IMB-6G-treated HCC cells. Bip is known as an ER stress marker protein [23], whereas CHOP is involved in ER stress-dependent apoptosis [24]. Immunoblotting analysis showed concentration- and time-dependent increases in the expression of Bip and CHOP proteins after treatment with IMB-6G in HepG2 and SMMC7721 cells (Figure 3A and 3B). To investigate whether Bip and CHOP proteins transcriptionally upregulated by IMB-6G, we examined the effects of IMB-6G on Bip and CHOP mRNA expression levels. Real-time PCR revealed that IMB-6G dose-dependently increased Bip and CHOP mRNA levels in HepG2 and SMMC7721 cells (Figure 3C and 3D). To further demonstrate ER stress is involved in IMB-6G-induced cell death, we used Salubrinal, an ER stress inhibitor [25], to inhibit ER stress activation. Salubrinal significantly suppressed IMB-6G-induced HepG2 cell death (Figure 3E) and apoptosis (Figure 3F). The percentage of cell viability in IMB-6G-treated HepG2 cells increased to 66.2 ± 5.0% with salubrinal pretreatment, compared to 34.3 ± 3.7% in DMSO pretreatment cells (*p* < 0.01) (Figure 3E); while the apoptotic cell death decreased from 41.9 ± 3.8% to 17.3 ± 2.9% (*p* < 0.01) (Figure 3F). These results collectively suggest the involvement of ER stress activation in the cytotoxicity of IMB-6G.

### IMB-6G activates ER stress pathway involving IRE1α and PERK

**Figure 4:**
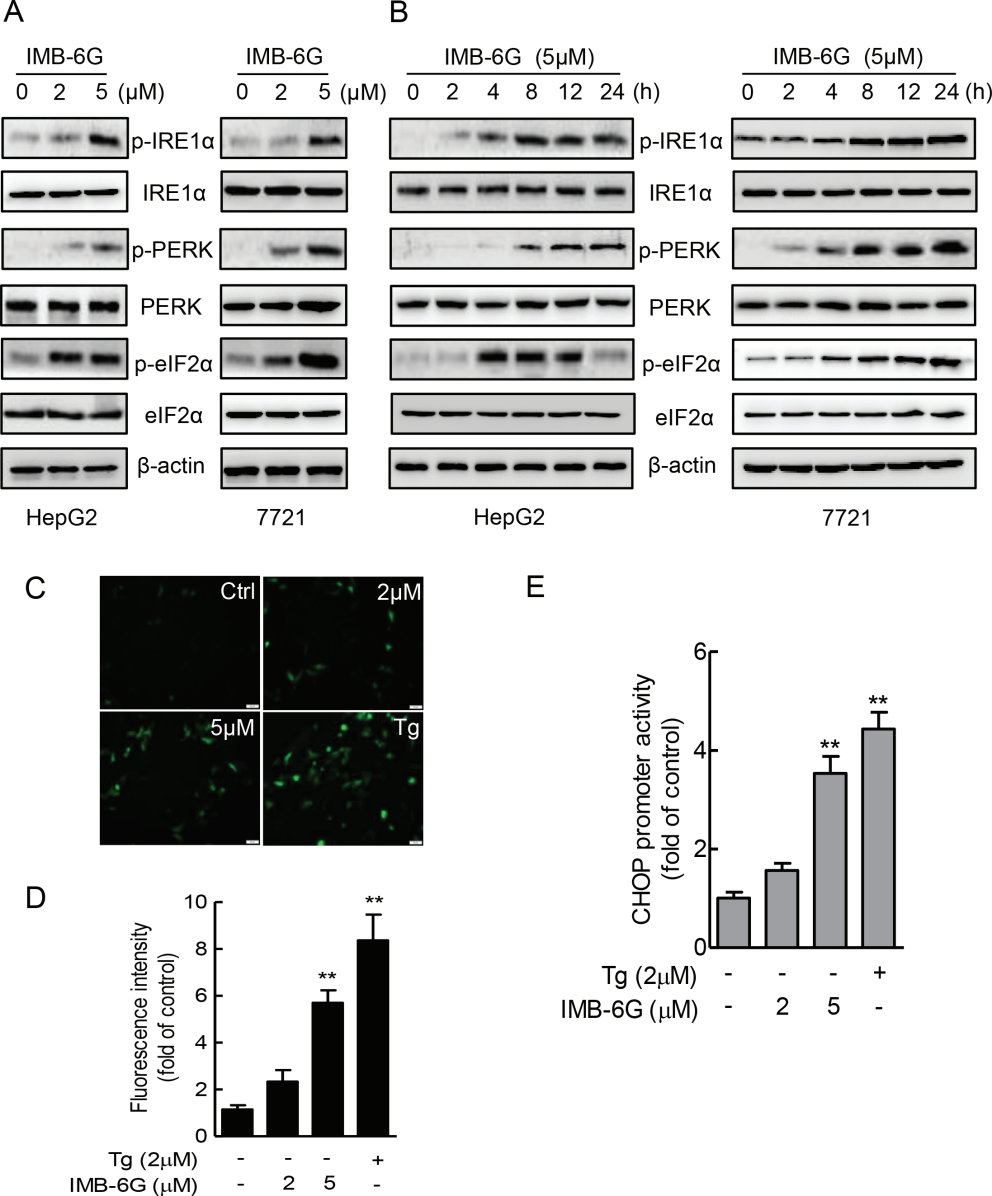
IMB-6G activates ER stress pathway involving IRE1α and PERK (**A** and **B**) HepG2 and SMMC7721cells were treated with IMB-6G at the indicated concentrations for 24 h (A), or treated with IMB-6G (5 μM) for indicated time points (B), the phosphorylation of IRE1α (Ser724), PERK (Thr980), eIF2α (Ser51) and expression levels of IRE1α, PERK and eIF2α were measured by immunoblotting using corresponding antibodies. (**C**) HepG2 cells were transfected with pCAX-F-XBP1ΔDBD-venus reporter plasmid for 24 h, followed by treatment with IMB-6G, Tg (positive control) or DMSO at the indicated concentrations for 6 h. The fluorescence images are visualized by fluorescence microscopy (scale bars, 20 μm). (**D**) Statistical analysis results of Figure 4C. (**E**) HepG2 cells were transfected with pGL3-CHOP promoter (−954/+91) vector and the pRL-TK vector as an internal control for 24 h, followed by treatment with IMB-6G, Tg, or DMSO at the indicated concentrations for 8 h. The level of luciferase activity was measured and normalized to Renilla luciferase activity. **p < 0.01 compared with untreated control group.

ER stress induces autophosphorylation of the cytoplasmic kinase domain of PERK and IRE1α as well as cleavage of p90ATF6 to p50ATF6 and initiates UPR [24, 26]. Thus, to identify ER stress signaling pathways that are activated by IMB-6G, we treated HepG2 and SMMC7721 cells with IMB-6G and examined the phosphorylation of PERK (Thr 980), IRE1α (Ser724) and the expression level of non-cleaved p90ATF6. Immunoblotting revealed that IMB-6G treatment increased the phosphorylation levels of IRE1α (Ser724) and PERK (Thr 980) in a concentration- and time-dependent manner in HCC cells (Figure 4A and 4B, upper panel), but did not increase the expression level of cleaved p50ATF6 (Supplementary Figure S3). Moreover, IMB-6G treatment increased the phosphorylation level of eIF2α (Ser51), which was phosphorylated by activated PERK (Figure 4A and 4B, lower panel). These results suggested that IRE1α and PERK ER stress pathways, but not ATF6 pathway, were activated by IMB-6G.

To further confirm that IMB-6G activates IRE1α and PERK pathways, the transcriptional activities of X-box protein 1 (XBP1) and CHOP were measured by reporter gene assay. pCAX-F-XBP1ΔDBD-venus reporter was a specific ER stress indicator based on XBP-1 mRNA splicing by IRE1α [27], the green fluorescence detected in cytoplasm indicates cell experiencing ER stress. pGL3-CHOP reporter plasmid containing CEBP-ATF composite site and ERSE element, which are involved in ER stress induction of CHOP [28]. HepG2 cells were transfected with pCAX-F-XBP1ΔDBD-venus or pGL3-CHOP reporter plasmid in the presence of DMSO, IMB-6G, or Thapsigargin (Tg), which is an ER stress inducer, and ER stress were monitored by fluorescence images or luciferase activity. In cells transfected with the pCAX-F-XBP1ΔDBD-venus reporter, IMB-6G and Tg treatment resulted in detectable fluorescence in the cells, whereas little was detected in DMSO control (Figure 4C and 4D). At the same time, IMB-6G and Tg treatment also significantly increased CHOP luciferase activity compared with DMSO control (Figure 4E). Taken together, these data suggest that IRE1α and PERK pathway might participate in the mechanisms mediating IMB-6G-induced apoptosis.

### IRE1α and PERK pathways are critical for IMB-6G induces apoptosis in HCC cells

**Figure 5:**
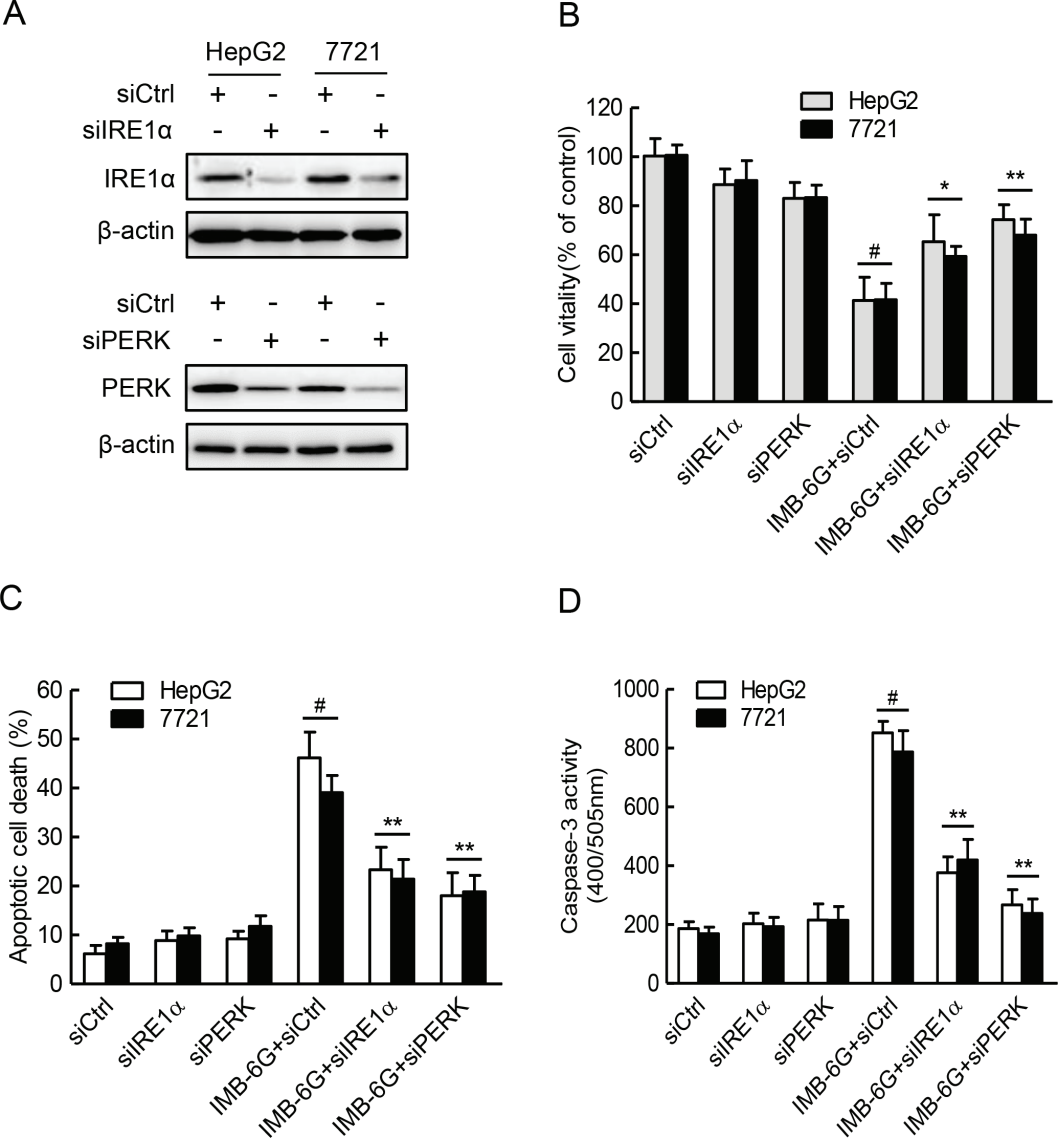
IRE1α and PERK pathways are critical for IMB-6G induced HCC cell death and apoptosis (**A**) HepG2 and 7721 cells were transfected with the control siRNA (siCtrl) or IRE1α siRNA (siIRE1α) or PERK siRNA (siPERK) for 48 h. Whole cell lysateswere subjected to immunoblotting analysis of IRE1α (upper panel), PERK (lower panel) and β-actin (as a loading control). (**B–D**) HepG2 and 7721 cells were transfected with control or IRE1α siRNA or PERK siRNA for 24 h, followed by treatment with IMB-6G (5 μM) for 24 h. Cell viability was assayed by MTT assay (B), the apoptotic rate was determined by flow cytometry (**C**), and caspase-3 activity (D) was determined as described in the materials and methods. ^#^*p* < 0.01 compared with siCtrl group, *p < 0.05, **p < 0.01 compared with IMB-6G-treated control group.

Having established that IMB-6G activation of the IRE1α and PERK pathways in HCC cells with unmitigated ER stress, we examined whether this plays a role in IMB-6G-induced apoptosis in HCC cells by knockdown of the expression of IRE1α or PERK (Figure 5A). Both HepG2 and 7721 cells with IRE1α or PERK knocked down displayed marked decrease in IMB-6G-induced cell death and apoptosis in comparison with those transfected with the control siRNA (Figure 5B and 5C). HCC cell death was reflected by cell viability loss (Figure 5B), while cell apoptosis was reflected by change of number of Annexin V staining positive cells (Figure 5C). Furthermore, a caspase-3 activity assay revealed that there were markedly reduced IMB-6G-induced caspase-3 activity in IRE1α or PERK knocked down HCC cells (Figure 5D), which is consistent with its decline of IMB-6G-induced apoptosis (Figure 5C). These results thus indicated that activation of IRE1α and PERK pathways play a critical role in IMB-6G-induced apoptosis in HCC cells.

### IRE1α-mediated ASK1-JNK activation contributes to IMB-6G-induced apoptosis

**Figure 6:**
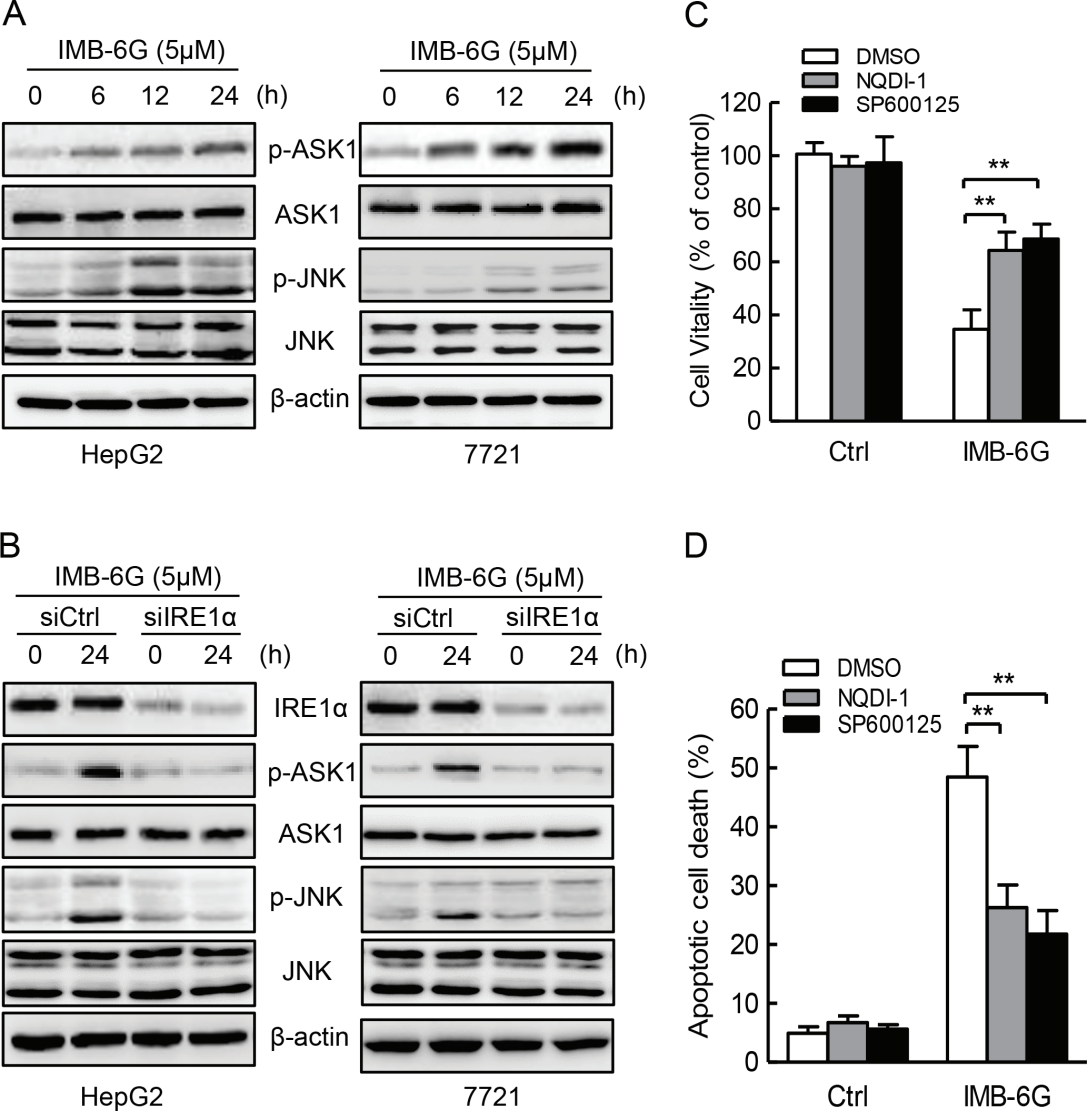
IMB-6G induces ASK1-JNK1 activation in HCC cells (**A**) HepG2 and SMMC7721cells were left untreated or treated with 5 μM IMB-6G for the indicated time points, the phosphorylation of ASK1(Thr845) and JNK (Thr183/Tyr185), total levels of ASK1 and JNK were determined by immunoblotting using specific antibodies. (**B**) HepG2 and SMMC7721cells were transfected by IRE1α siRNA or control siRNA for 24 h, followed by 5 μM IMB-6G treatment for 24 h, the phosphorylation of ASK1 (Thr845) and JNK (Thr183/Tyr185), total levels of IRE1α, ASK1 and JNK were determined by immunoblotting. (**C and D**) HepG2 cells were pretreated with ASK1 specific inhibitor NQDI-1(10 μM) or JNK specific inhibitor SP600125 (20 μM) for 1 h, followed by IMB-6G (5 μM) treatment for 24 h. Cell viability was analyzed by MTT assay (C), and the apoptotic rate was determined by flow cytometry (D). **p < 0.01 compared with IMB-6G-treated control group.

Previous studies have shown that IRE1α activation contributes to ER stress-induced cell apoptosis through activation of ASK1-JNK signaling [29]. Since IMB-6G induced the phosphorylation of IRE1α (Figure 4A and 4B), and IRE1α silencing suppressed IMB-6G-induced HCC cell death and apoptosis (Figure 5B and 5C), we thus examined whether IMB-6G induces the activation of ASK1 and JNK through IRE1α in HCC cells. Firstly, Immunoblotting results showed that IMB-6G treatment induced a significant ASK1 (Thr845) and JNK (Thr183/Tyr185) phosphorylation in HepG2 and SMMC7721 cells (Figure 6A). In addition, siRNA knockdown of IRE1α markedly inhibited the phosphorylation of ASK1 (Thr845) and JNK1 (Thr183/Tyr185) in IMB-6G-treated HCC cells (Figure 6B). Moreover, the specific ASK1 inhibitor (NQDI-1) and JNK inhibitor (SP600125) suppressed IMB-6G-induced HepG2 cell death (Figure 6C) and apoptosis (Figure 6D), as the percentage of cell viability increased dramatically and the number of apoptotic cell death decreased significantly with ASK1 or JNK inhibition. These results together suggest that IMB-6G induces ER stress to activate ASK1-JNK signaling, which mediates apoptosis in HCC cells.

### PERK-CHOP signaling plays a key role in IMB-6G induced apoptosis

**Figure 7:**
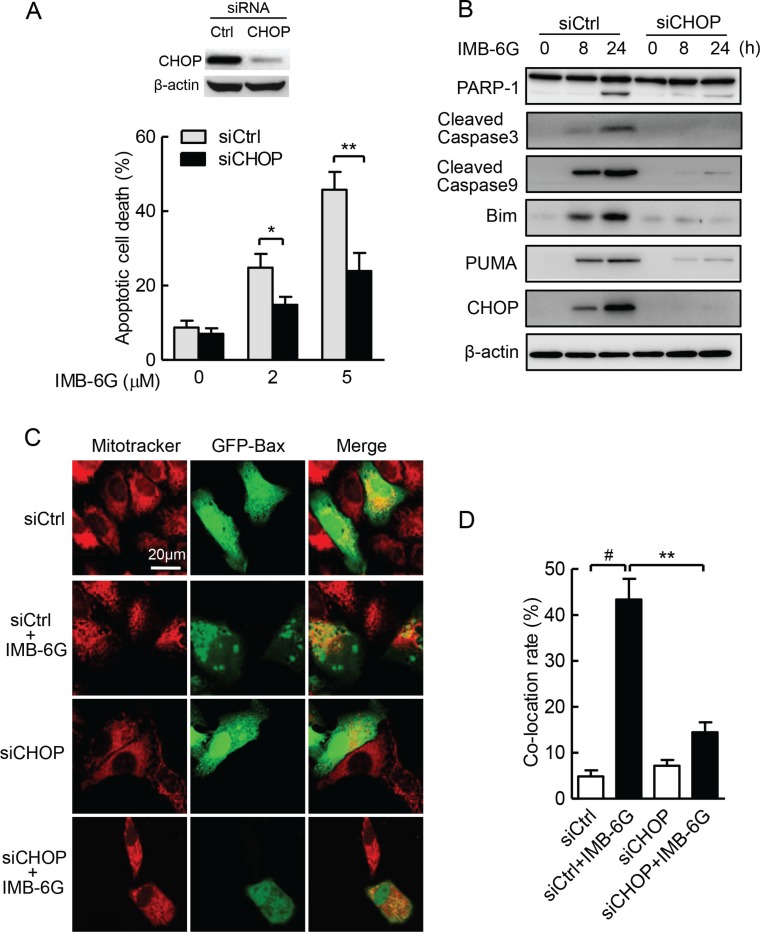
ER stress-induced CHOP plays a key role in IMB-6G-induced apoptosis (**A**) HepG2 cells were transfected with siCtrl or siCHOP for 24 h, followed by treatment with 2 μM or 5 μM IMB-6G for 18 h. Cells were stained with Annexin V-FITC/PI, the apoptotic cells were analyzed by flow cytometry. Knockdown of CHOP by siRNA was measured by immunoblotting. *p < 0.05, **p < 0.01compared with siCtrl group. (**B**) HepG2 cells were transfected with siCtrl or siCHOP for 24 h, followed by treatment with 5 μM IMB-6G for the indicated time. The expression levels of PARP-1, cleaved caspase-3 and 9, Bim, PUMA and CHOP were determined by immunoblotting with the indicated antibodies. (**C**) HepG2 cells were transfected GFP-Bax plasmid along with siCHOP or siCtrl for 36 h, followed by 5 μM IMB-6G treatment for 12 h. The effect of CHOP knockdown on Bax translocation to mitochondria was visualized by confocal microscope as described in Figure 2B. (**D**) Percentage of Bax translocation was determined by counting three different fields (20–40 cells/field), and the statistical analysis result is shown. ^#^*p* < 0.01 compared with siCtrl group; **p < 0.01 compared with IMB-6G-treated siCtrl group.

Numerous studies show that CHOP is a main driver of ER stress-associated apoptosis via the PERK-eIF2α pathway [4, 30]. Because IMB-6G significantly increased the phosphorylation of PERK (Figure 4) and CHOP expression in HCC cells (Figure 3), meanwhile PERK silencing suppressed IMB-6G-induced HCC cell death and CHOP expression (Figure 5 and Supplementary Figure S4), we next investigated whether CHOP induction is essential for IMB-6G-induced apoptosis. siRNA knockdown of CHOP expression significantly suppressed IMB-6G-induced HepG2 cell apoptosis (Figure 7A). In addition, CHOP siRNA markedly reduced the levels of cleaved PARP-1, caspase-3 and caspase-9 in IMB-6G-treated HepG2 cells as compared with control siRNA (Figure 7B, upper panel). Since IMB-6G upregulated the expression levels of BH3-only proteins Bim and PUMA (Figure 2D), we next investigated whether knockdown of CHOP decreases the expression levels of Bim and PUMA in IMB-6G-treated HCC cells. Immunoblotting results indicated that the expression levels of Bim and PUMA were significantly decreased when HepG2 cells were treated with CHOP siRNA in the presence of IMB-6G (Figure 7B, middle panel).

The fact that IMB-6G induced Bax translocation into mitochondria and initiates apoptosis (Figure 2A and 2B) compelled us to examine whether CHOP is required for IMB-6G-induced Bax translocation. The effects of CHOP knockdown on translocation of Bax into the mitochondria induced by IMB-6G is clearly shown in Figure 7C. In siRNA control cells, IMB-6G treatment triggered Bax accumulation in mitochondria. However, CHOP knockdown in HepG2 cells efficiently suppressed IMB-6G-induced Bax accumulation in mitochondria. The statistical analysis of the percentage values of co-localization is also shown in Figure 7D. Taken together, these observations indicate that CHOP signaling is the key factor of IMB-6G-induced apoptosis in HCC cells.

## DISCUSSION

In this study, we have provided evidences that IMB-6G-induced apoptosis of human HCC cells is mediated by ER stress response. To be noted, the possible inducing effect of IMB-6G on apoptosis in tumor cells was reported in our previous report [18]. However, only in the present study, IMB-6G inducing ER stress-mediated apoptosis was first reported and studied in depth.

IMB-6G induced activation of two branches of ER stress and related signal proteins. In HCC cells incubated with IMB-6G, increase in expression of Bip was observed. In addition, eIF2α and CHOP, markers of activation of PERK branch of ER stress [4], were activated by IMB-6G. Meanwhile, the activation of IRE1α and its downstream protein ASK1 and JNK [31] were also induced by IMB-6G. Especially, the activation of CHOP and XBP1 suggested that IMB-6G might induce transcription of ER stress target genes, such as Bim and PUMA. Moreover, the fact that pretreatment of Salubrinal, an ER stress inhibitor, attenuates IMB-6G-induced apoptosis confirmed the role of ER stress in IMB-6G-induced cell death.

It has been reported that IRE1α is required for ER stress-induced apoptosis through the formation of an IRE1α-TRAF2-ASK1 complex [31]. Consistent with this, we found that IMB-6G-induced ER stress was associated with persistent activation of IRE1α signaling. This was demonstrated by the sustained presence of phosphorylated IRE1α, ASK1 and JNK, and decreased ASK1 and JNK activation by the siRNA knockdown of IRE1α. Importantly, selective disruption of the IRE1α-ASK1-JNK axis of ER stress by the specific ASK1 or JNK inhibitor following IMB-6G treatment reduced the apoptosis in HCC cells, indicating the critical role of activation of IRE1α-ASK1-JNK signaling in response to IMB-6G treatment.

Activation of CHOP downstream of PERK signaling plays an important role in executing ER stress-induced apoptosis in diverse types of cells [6]. In line with this, our results showed IMB-6G induced an increase in both mRNA level and protein level of CHOP, and silencing of CHOP by siRNA suppressed IMB-6G-induced apoptotic cell death, indicating that CHOP is an important mediator of IMB-6G-elicited cell death. This finding led us to further study the translocation of Bax into mitochondria in cells treated with IMB-6G, since it has been reported that CHOP expression is able to induce Bax translocation and apoptosis [32]. Consistent with the increase in CHOP level, translocation of Bax into mitochondria was observed in IMB-6G-treated cells, and silencing of CHOP by siRNA efficiently suppressed IMB-6G-induced Bax accumulation in mitochondria. These results suggested the involvement of CHOP and mitochondrial-dependent Bax translocation in the apoptosis induced by IMB-6G. However, Bax translocation independent of CHOP had also been reported before [33, 34]. The possibility of involvement of other factors in Bax translocation induced by IMB-6G could not be excluded.

The BH3-only proteins are essential initiators of apoptosis by exerting their ability to bind and inhibit pro-survival Bcl-2 members [35]. Our results indicated that the expression levels of Bim and PUMA were increased by IMB-6G. Bim and PUMA are direct link between ER stress and apoptosis and are essential for ER stress-induced apoptosis in a diverse range of cell types [36, 37]. The expression of Bim as well as PUMA was reported to be increased by CHOP [9, 38]. Our results also showed that siRNA for CHOP could significantly reduce IMB-6G-induced Bim and PUMA expression. Therefore, the sequential links between ER stress, CHOP, Bim, PUMA and Bax translocation might indicate a clear pathway of mitochondrial apoptosis induced by IMB-6G.

In conclusion, here we show that IMB-6G is a potent antitumor agent that acts via induction of ER stress-associated apoptosis in HCC cells. IMB-6G triggered activation of IRE1α-ASK1 and PERK-CHOP-mediated ER stress, increased transcription of ER stress target genes such as Bim and PUMA, induced Bax translocation into mitochondria and finally activated mitochondrial-dependent apoptosis. Targeting ER stress by IMB-6G may thus provide a novel therapeutic option in the treatment of HCC.

## MATERIALS AND METHODS

### Reagents

IMB-6G was synthesized according to previously described methods [18]. Antibodies against Bip, CHOP, PERK, IRE1α, eIF2α, phospho-PERK (Thr980), phospho-eIF2α (Ser51), phospho-ASK1 (Thr845), phospho-JNK (Thr183/Tyr185), Bax and Bad were purchased from Cell Signaling Technology (Danvers, MA, USA). Anti-phospho-IRE1α (Ser724) and ATF6 antibodies were from Abcam (Cambridge, MA, USA). Anti-Bim, PUMA and Cytochrome c antibodies were purchased from Santa Cruz (Santa Cruz, CA, USA). SP600125, NQDI-1, Thapsigargin, MTT and β-actin antibody were obtained from Sigma (St. Louis, MO, USA). Salubrinal (ER stress inhibitor) was purchased from Selleckchem (Shanghai, China).

### Cell culture and cell viability assay

Human HCC cell line HepG2 and SMMC7721 were purchased from Shanghai Institutes for Biological Sciences of Chinese Academy of Sciences. All cells were cultured in Dulbecco's modified Eagle's medium (Hyclone, UT, USA) supplemented with 10% fetal bovine serum (Hyclone, UT, USA), 100 U/ml penicillin, and 100 μg/ml streptomycin sulfate and incubated at 37°C in a humidified atmosphere with 5% CO_2_. The effect of IMB-6G on the cell viability of HepG2 or SMMC7721 cells was evaluated by MTT assay as described previously [39].

### Flow cytometric analysis of apoptosis and caspase-3 activity assay

Detection of apoptotic cells induced by IMB-6G was performed using Annexin V-FITC/PI apoptosis detection kit (KeyGen, Nanjing, China) according to the manufacturer's instructions. Briefly, cells were harvested and washed with PBS twice, incubated with Annexin V-FITC and PI for 30 min in the dark. After that, the fluorescence of each sample was quantitatively analyzed by FACSCalibur flow cytometer and CellQuest software (BD Biosciences, Sparks Glencoe, MD, USA). The caspase-3 activity induced by IMB-6G was detected as previously reported [40].

### Isolation of mitochondria and cytoplasm fractions

Cell Mitochondria Isolation kit (Beyotime, Shanghai, China) was used to prepare mitochondria and cytoplasm fractions according to the manufacturer's instructions. Briefly, 2 × 10^7^ cells were collected using a cell scraper and then washed with PBS buffer. After centrifugation, cells were resuspended in 1 mL mitochondria isolation buffer. After incubated for 10 min on ice, cells were homogenized using a Wheaton Dounce Tissue Grinder (4 m/s, 15 s, 3 times). Then the pellet (mitochondria fraction) and the supernatant (cytoplasm fraction) were isolated by centrifugation. The mitochondria fraction was resolved with 2% CHAPS in Tris-buffered saline (25 mM Tris, 0.15 M NaCl, pH 7.2).

### Transient transfection and luciferase assay

HepG2 or SMMC7721 cells were co-transfected with pCAX-F-XBP1ΔDBD-venus or pGL3-CHOP plasmid plus the pRL-TK plasmid using the Vigofect transfection reagent (Vigorous, Beijing, China) as instructed by the manufacturers. After 24 hours of transfection, cells were pretreated with the indicated concentrations of IMB-6G for 6~8 hours. The fluorescence images are visualized by fluorescence microscopy (Axio VertA1, Zeiss, Germany). For luciferase assay, the cells were lysed, and the luciferase activity was determined using the Dual-luciferase reporter assay system (Promega, Madison, CA, USA) as described previously [39].

### Real-time quantitative PCR

Total messenger RNA from HepG2 or SMMC7721cells was isolated by Trizol reagent (Invitrogen, CA, USA). First-strand cDNA synthesis and PCR reaction were conducted as described before [39]. Total RNA was normalized in each reaction using GAPDH cDNA as an internal standard. The primers of target genes were as follows: Bip (sense 5′-TAGCGTA TGGTGCTGCTGTC-3′, anti-sense 5′-TTTGTCAGGGG TC TTTCACC-3′); CHOP (sense 5′-GAGGAGAGAGTG TTCAAGAAGG-3′, anti-sense 5′-TCTGGGAGGTGC TTGTGAC-3′), GAPDH (sense 5′-CTCAGACACCAT GGG GAAGGTGA-3′, anti-sense 5′-ATGATCTTGAGG CTGTTGTCATA-3′).

### Small RNA interference (siRNA) and immunoblotting analysis

Specific siRNAs for IRE1α (5′-GCGUCUUUUACU ACGUAAUCU-3′) [41], CHOP (5′-AAGAACCAGCAG AGGUCACAA-3′) [42] and a non-targeting control siRNA were synthesized by Shanghai Gene Pharma (Shanghai, China). PERK siRNA (sc-36213) was purchased from Santa Cruz Biotechnology (SantaCruz, CA). The transfection of siRNA in HepG2 or SMMC7721 cells were carried out with Lipofectamine RNAiMAX reagent (Invitrogen, Carlsbad, CA) according to the manufacturer's instructions. Twenty-four hours after transfection, the cells were treated with or without IMB-6G for the indicated time point before the subsequent experiments. Immunoblotting was performed as described previously [39, 43].

### Measurement of Bax translocation into mitochondria

The influence of IMB-6G on Bax translocation was examined using cells transfected with GFP-Bax plasmid. To check the effects of CHOP on the translocation of Bax, cells were co-transfected GFP-Bax with siCHOP or siControl, followed by IMB-6G treatment. After that, cells were stained with mitochondria probe Mitotracker M7512 solution (100 nM) for 30 min at 37°C. After PBS washing, cells were fixed in 4% paraformaldehyde for 15 min at 37°C. The fluorescence of GFP-Bax (green) and Mitotracker M7512 (red) were observed with a Zeiss confocal scanning microscope. Quantification of colocalization of the two labels (green and red) was conducted using the ‘Colocalization’ module of Image Pro plus 6.0.

### Statistical analysis

Results are presented as mean values ± standard error of independent triplicate experiments. All statistical analyses were performed by using ANOVA and Student's *t*-test, and *P*-values of less than 0.05 were considered statistically significant.

## SUPPLEMENTARY MATERIALS FIGURES



## Abbreviations

HCC: hepatocellular carcinoma
ER: endoplasmic reticulum
UPR: unfolded protein response
PERK: pancreatic endoplasmic reticulum eIF2a kinase
IRE1α: inositol requiring enzyme 1α
ATF6: activating transcription factor 6
CHOP: C/EBP homologous protein
ASK1: apoptosis signal-regulating kinase 1
JNK: c-Jun N-terminal kinase
PARP-1: poly (ADP-ribose) polymerase 1.
