# Supplementary material for: IMB-6G, a novel N-substituted sophoridinic acid derivative, induces endoplasmic reticulum stress-mediated apoptosis via activation of IRE1α and PERK signaling
Source: Oncotarget. 2016 Mar 18;7(17):23860–73. doi: 10.18632/oncotarget.8184 (PMC5029669; doi:10.18632/oncotarget.8184)
Supplement: Supplementary file 1 [file oncotarget-07-23860-s001.pdf]

IMB-6G, a novel *N*-substituted sophoridinic acid derivative, induces endoplasmic reticulum stress-mediated apoptosis *via* activation of IRE1α and PERK signaling

Supplementary Materials

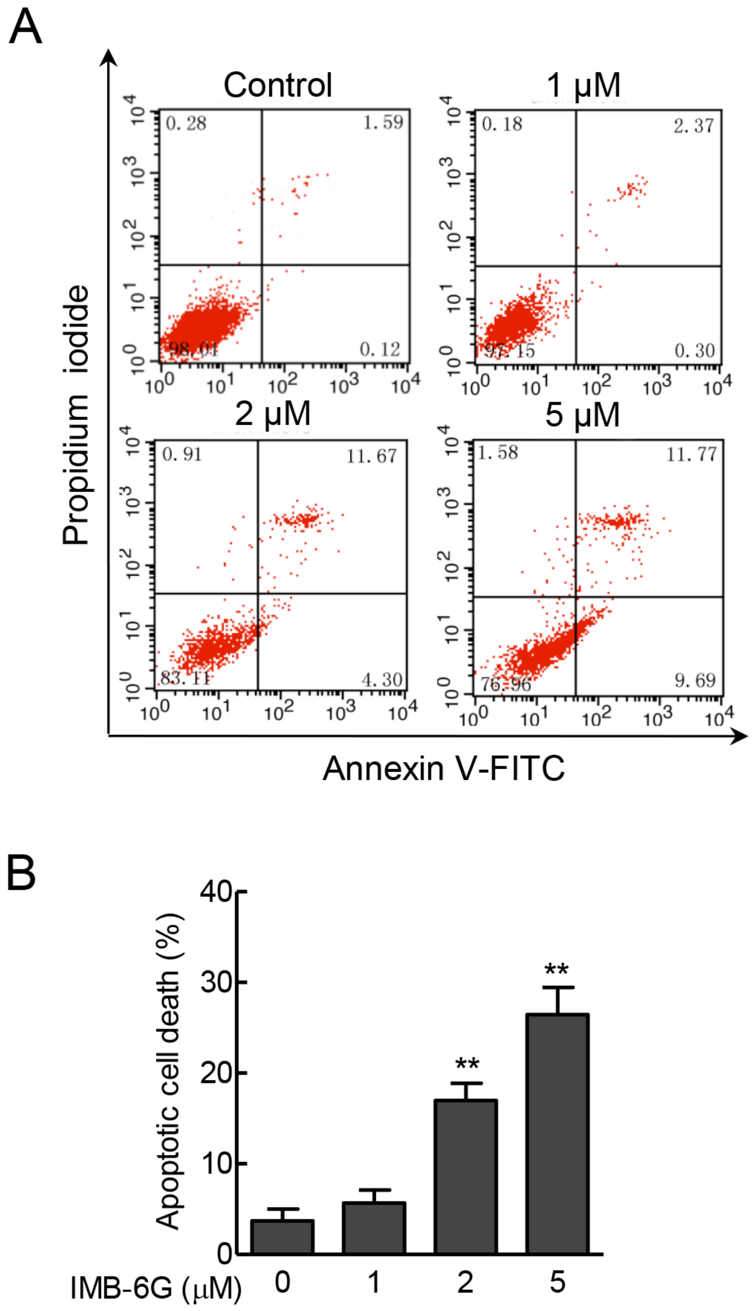

Supplementyary Figure S1: IMB-6G induces apoptosis in SMMC7721 cells. (**A**) SMMC7721 cells were stained with Annexin V-FITC and PI after incubated with indicated concentrations of IMB-6G for 24 h, the numbers of apoptotic cells were analyzed by flow cytometry. (**B**) Statistical analysis result of flow cytometric analysis of apoptosis. Annexin V-positive cells were accepted as apoptotic cells. The results are presented as mean ± standard error and represent three individual experiments. $**p < 0.01$ compared with untreated control group.

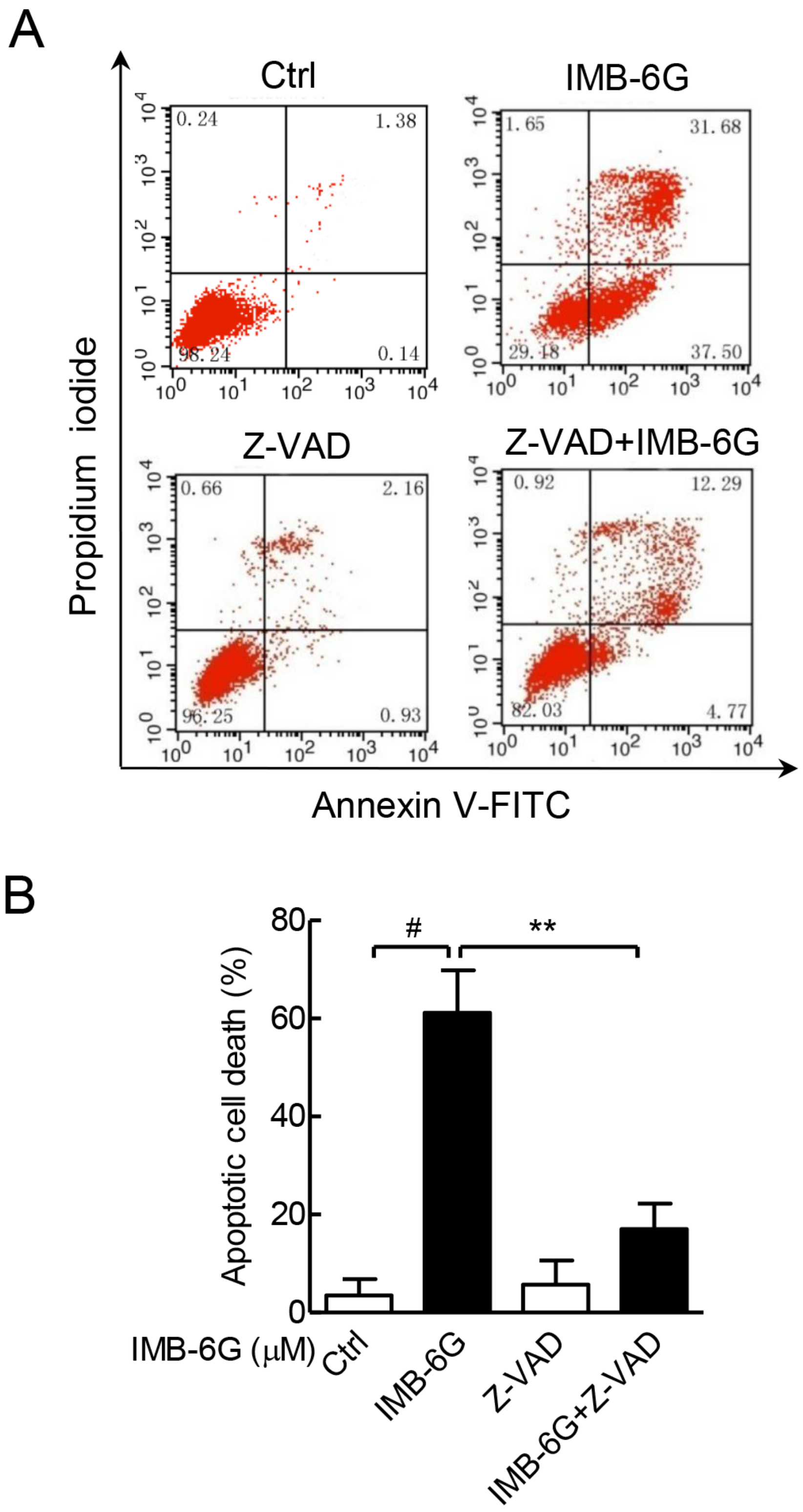

Supplementyary Figure S2: Z-VAD inhibits IMB-6G induces apoptosis in HepG2 cells. (**A**) HepG2 cells were incubated with 10 μM Z-VAD or vehiculum control (DMSO) for 1 h, followed by IMB-6G treatment for 24 h. Then cells were stained with Annexin V-FITC and PI and the numbers of apoptotic cells were analyzed by flow cytometry. (**B**) Statistical analysis result of flow cytometric analysis of apoptosis. Annexin V-positive cells were accepted as apoptotic cells. The results are presented as mean ± standard error and represent three individual experiments. $^{\#}p < 0.01$ compared with control group, $^{**}p < 0.01$ compared with IMB-6G-treated group.

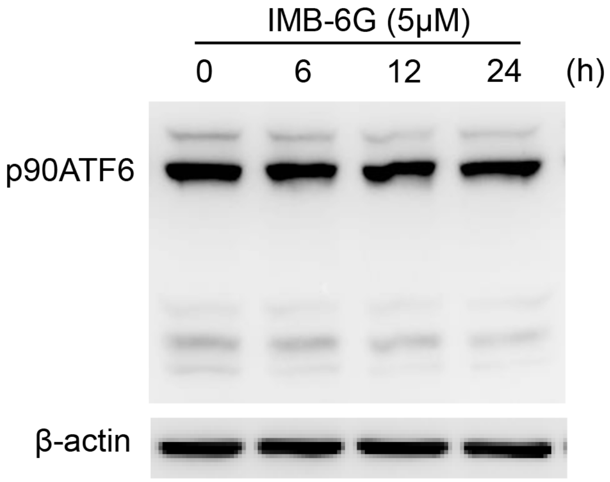

Supplementyary Figure S3: Effects of IMB-6G on the expression level of ATF6. HepG2 cells were treated with 5 μM IMB-6G for the indicated time points, the expression level of non-cleaved p90ATF6 and β-actin were measured by immunoblotting.

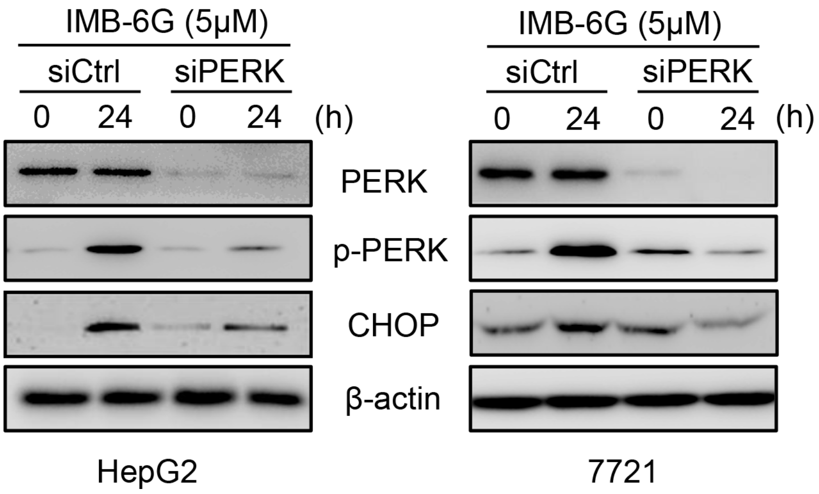

Supplementyary Figure S4: Silencing PERK suppressed IMB-6G-induced CHOP expression. HepG2 and SMMC7721cells were transfected by PERK siRNA or control siRNA for 24 h, followed by 5 μM IMB-6G treatment for 24 h, the phosphorylation of PERK, total levels of PERK and CHOP were determined by immunoblotting.
